# *Nectandra grandiflora* By-Products Obtained by Alternative Extraction Methods as a Source of Phytochemicals with Antioxidant and Antifungal Properties

**DOI:** 10.3390/molecules23020372

**Published:** 2018-02-09

**Authors:** Daniela Thomas da Silva, Rene Herrera, Berta Maria Heinzmann, Javier Calvo, Jalel Labidi

**Affiliations:** 1 Center of Rural Sciences, Federal University of Santa Maria, Ave. Roraima 1000, Santa Maria 97105-900, Brazil; dthomasdasilva@gmail.com; 2 Biorefinery Processes Research Group, Department of Chemical and Environmental Engineering, University of the Basque Country (UPV/EHU), Plaza Europa 1, 20018 Donostia, Spain; renealexander.herrera@ehu.eus; 3 Department of Industrial Pharmacy, Federal University of Santa Maria, Ave. Roraima 1000, Santa Maria 97105-900, Brazil; berta.heinzmann@gmail.com; 4 Chromatography and Mass Spectrometry Platform, CIC BiomaGUNE, Paseo Miramon 182, 20009 San Sebastian, Spain; jcalvo@cicbiomagune.es

**Keywords:** forest residues, phenolic compounds, natural antioxidants, quercitrin, value-added by-products

## Abstract

*Nectandra grandiflora* Nees (Lauraceae) is a Brazilian native tree recognized by its durable wood and the antioxidant compounds of its leaves. Taking into account that the forest industry offers the opportunity to recover active compounds from its residues and by-products, this study identifies and underlines the potential of natural products from *Nectandra grandiflora* that can add value to the forest exploitation. This study shows the effect of three different extraction methods: conventional (CE), ultrasound-assisted (UAE) and microwave-assisted (MAE) on *Nectandra grandiflora* leaf extracts (NGLE) chemical yields, phenolic and flavonoid composition, physical characteristics as well as antioxidant and antifungal properties. Results indicate that CE achieves the highest extraction phytochemical yield (22.16%), but with similar chemical composition to that obtained by UAE and MAE. Moreover, CE also provided a superior thermal stability of NGLE. The phenolic composition of NGLE was confirmed firstly, by colorimetric assays and infrared spectra and then by chromatographic analysis, in which quercetin-3-*O*-rhamnoside was detected as the major compound (57.75–65.14%). Furthermore, the antioxidant capacity of the NGLE was not altered by the extraction methods, finding a high radical inhibition in all NGLE (>80% at 2 mg/mL). Regarding the antifungal activity, there was observed that NGLE possess effective bioactive compounds, which inhibit the *Aspergillus niger* growth.

## 1. Introduction

Innovative and environmental-friendly approaches are the key to increase the profitability, economic viability and sustainability in the forest industry by optimizing the process in order to obtain high-valued products (bio/chemicals and biomaterials). Forest residues (bark, foliage, branches) represent a renewable feedstock that has been used for many years as a combustible material, however, the development of by-products is an essential path for forest valorization [1]. Tree bark and foliage constitute a little explored but promising source of natural compounds or phytochemicals (in the form of pure or as mixtures/extracts) that could be used as active ingredients for agronomic, cosmetic, food additives, pharmaceutical and in nutraceutical formulations [1,2]. Several techniques have been described for extracting active natural compounds from low-cost raw material [3]. These procedures include the so-called heating systems, such as traditional Soxhlet and heat reflux extraction [4,5], ultrasound-assisted extraction [6,7] and microwave-assisted extraction [8,9], as well as supercritical fluid and pressurized extraction [10,11] or the combination of these extraction techniques [12].

Conversely, many natural matrix products are thermally unstable and may degrade under thermal extraction conditions [12]. Moreover, large consumption of solvents, energy and lengthy extraction time are some drawbacks that should also be taken into account. The ideal extraction procedure has to retain the maximum of the bioactive constituents in a shortest processing time with low economic costs [13] and low environmental impact [14]. Additionally, the extraction methods should be simple, safer for users and with a level of automation for industrial application [14,15]. In general, the selection of an appropriate extraction procedure depends on the type of compound to be extracted, as well as the development of the technique [16]. Several studies reported the efficiency of microwave-assisted extraction (MAE) and ultrasound-assisted extraction (UAE) for increasing the content of polyphenols [9,17]. In MAE, the microwave energy is used to heat the polar solvents in contact with solid samples and thus, recovering the target compounds [18]. Likewise, the UAE involves a superficial disrupt of plant tissue, allowing the penetration of solvent into cell walls through the acoustic cavitation [19].

It is worth noting that the Brazilian flora is a rich source of phytochemicals, aromas and bioactive compounds of medicinal and pharmaceutical importance, as well the fine chemicals segment [20]. *Nectandra* is one of the largest genera of Lauraceous family that includes ca. 120 tree species and more than 190 reported different types of natural substances with several therapeutic applications [21]. *Nectandra grandiflora* Nees, commonly known as “canela-amarela” or “canela-fedida”, is a medium-sized tree (10–15 m) endemic of Brazilian Atlantic forest and Cerrado biomes [22]. This species presents a moderately heavy and naturally durable wood recommended for timbering and furniture [23]. On the other hand, there are not enough scientific studies regarding the environmental-friendly and cost-effective technologies to recover phytochemicals (e.g., phenolic compounds) from *Nectandra grandiflora* leaves. Ribeiro et al. [24] extracted flavonoid glycosides (natural antioxidants) and neoliganans from the tree foliage by conventional heating processing (Soxhlet). On these grounds, the present study aims to address the unexplored potential of *Nectandra grandiflora* co-products describing the phenolic composition, thermal behavior, antioxidant and antifungal properties of its leaf extracts obtained by alternative green processes (MAE and UAE).

## 2. Results and Discussion

### 2.1. Extraction Yields and Phytochemicals Contents

Extraction yield refers to the percentage of ethanolic extract obtained from a dried plant sample through an extraction technique [17]. The three extraction methods applied on *Nectandra grandiflora* leaves showed significantly different yields of phytochemicals (Table 1). 

The conventional Soxhlet (CE) method presented the highest yield (22.16 g DW/100 g dried plant), followed by ultrasound-assisted (UAE) and microwave-assisted extraction (MAE). The highest yield achieved by conventional extraction compared to ultrasound- and microwave-assisted methods can be explained by the application of heat for a longer period. However, the processing time used in ultrasound and microwave heating methods was significantly shorter (30 min) than for the conventional one and taking the energy consumption into account, UAE and MAE appear as favorable extraction methods for *Nectandra grandiflora* leaves. Our findings are in accordance with Mustapa et al. [25], who reported a superior yield of *Clinacanthus nutans* extracts by CE compared to MAE. According to Chirinos et al. [26], after 60 min, increasing extraction time did not significantly improve the phytochemical yield and may increasing the risk of phenolic oxidation (alterations in color, aroma and product quality).

In this work, the three evaluated extraction methods were able to recover high contents of total phenolic compounds, flavonoid and condensed tannins. However, we detected that the values determined in the extracts depended significantly on the process applied (Table 1). The CE extract presented higher values of total phenolic and flavonoid contents (279 mg GaE/g DW and 150.85 mg QE/g DW, respectively) than UAE and MAE extracts. Considering the composition of natural sources of polyphenols and flavonoid compounds, as well as their chemical structures and properties, an universal extraction procedure is not feasible and a specific method must be optimized for each natural bioactive compound [27,28]. Currently, some alternative techniques such as extraction under pressure (N_2_) or enzymatic extraction in combination with UAE and MAE have been applied to increase phenolic yields from plant matrices [29,30].

### 2.2. FTIR Analysis

**Figure 1:**
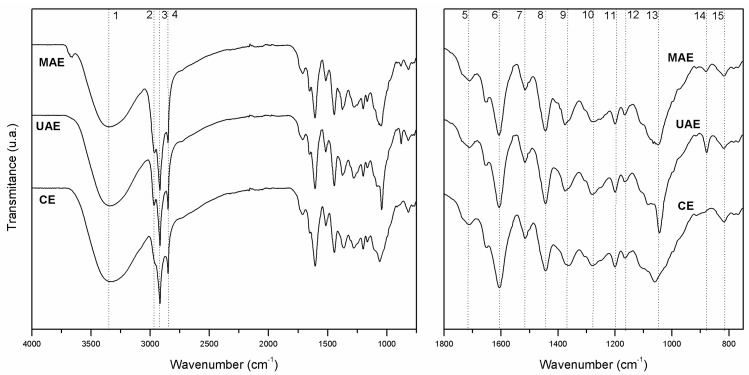
FTIR spectra of leaf extracts obtained from *Nectandra grandiflora*, by conventional Soxhlet extraction (CE), ultrasound-assisted extraction (UAE) and microwave-assisted extraction (MAE). Wavenumber range 4000–800 cm^−1^ (**Left**) and fingerprint region 1800–750 cm^−1^ (**Right**); band assignments are shown in Table S1.

Leaf extracts exhibited similar absorption bands in FTIR spectra but with slight differences in the extract obtained by CE. The spectra profiles are presented in Figure 1 and the assignments are given in Table S1.

Analysis of the FTIR spectra ranging from 3400 to 3200 cm^−1^ shows the sum of different vibrational bands of –OH groups. The elongated U shape around this region is characteristic of alcoholic and phenolic compounds [31,32]. The region of 2945–2845 cm^−1^ is composed by the overlapping of the CH_2_, and CH_3_ stretching asymmetric and symmetric vibrations; possibly derived from carbohydrates [33].

However, these first regions analysed do not present conclusive features to identify the nature of the phytochemicals. Several authors have described the FTIR spectra fingerprinting region (1800–750 cm^−1^) because the target functional groups appear primarily in this range [25]. The weak peak at 1709 cm^−1^ shows the presence of the carbonyl group, possibly due to dimeric saturated acids [31]. The signals detected in the range 1615–1440 cm^−1^ (peaks 6–8) are assigned to aromatic ring stretching vibrations. A strong and intense peak at 1606 cm^−1^ corresponds to within-ring skeletal stretching, alongside with the stretching of the C=C–C aromatic bond that appears at 1515 cm^−1^.

The peak in the region of 1375–1361 cm^−1^ is assigned to the hydroxyl in-plane bending of primary and secondary alcohols [31,34]. Furthermore, *Nectandra grandiflora* extracts also show bands in the 1277–1271 cm^−1^ region, which correspond to the C–O asymmetrical stretching vibration arising from the pyran-derived ring structure of flavonoids [33]. The peak around 1200 cm^−1^ is associated with phenol C–OH stretches.

The 1154–1046 cm^−1^ region (peak 13) can be assigned to the C–H in-plane deformation of aromatic compounds [33]. The extract obtained by UAE exhibited a strong and intense peak, while the other extracts only exhibit shoulders in this region. Finally, the aromatic C–H out-of-plane bending vibration region between 920 and 750 cm^−1^ mostly shows signals of low intensity [32]. The extract obtained by ultrasound technique shows a medium-intensity signal at 878 cm^−1^ corresponding to the deformation of the C–H bond in a substituted *meta*-diaromatic compound [35]. This signal was lower for the MAE extract and did not appear at all in the CE extract. Another low-intensity peak at 816 cm^−1^ can be seen in the FTIR spectra of all extracts.

The presence of peaks due to hydroxyl and carbonyl vibrations indicates that there are some polar compounds in the *Nectandra grandiflora* foliage extracts, such as flavonoids, neolignans and phenolic acids. These results are in agreement with those found by the total phenolic and flavonoid contents in this study and other scientific studies [24,36].

### 2.3. LC-UV/ESI-HR-MS and MALDI/MS/MS Analysis

In the LC-MS and MALDI/MS/MS analysis of *Nectandra grandiflora* leaf extracts, six compounds were detected based on their retention time, UV (wavelength of maximum absorbance) and mass spectra and MS fragmentation parameters. The molecular mass of the compounds was obtained from their positive ion electrospray mass spectra (ESI-MS), which showed the corresponding protonated pseudomolecular ions as well as the sodium adduct ions (parent ions). Table 2 lists the major (>5%) compounds detected in *Nectandra grandiflora* extracts. 

**Figure 2:**
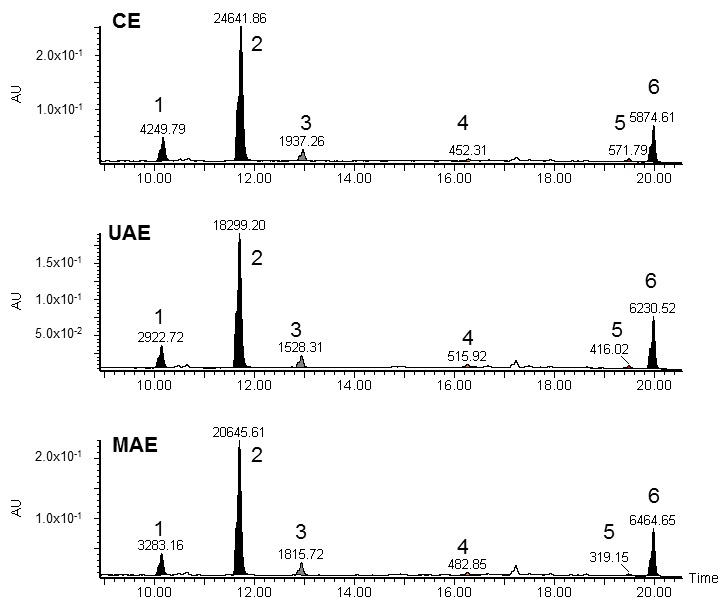
LC-UV chromatograms at 280 nm of the *Nectandra grandiflora* extracts obtained by conventional Soxhlet extraction (CE), ultrasound-assisted extraction (UAE) and microwave-assisted extraction (MAE). For peak identification, see Table 2.

All extracts presented a similar phenolic profile (Figure 2) with some differences in the estimated percentages of the compounds. More than 76% of the total chemical composition was established, achieving 81.71% in the conventional method. The compounds identified are glycosylated flavonols, of which quercetin rhamnoside (quercitrin) was the most abundant in the extract obtained by the conventional method (65.32%). Kaempferol rhamnoside (afzelin) (11.26–9.77%) and myricetin rhamnoside (myricitrin) (<6%) were also detected. Figure S2 shows the MS fragmentation of the peaks 1, 2 and 3 obtained through MALDI/MS/MS analysis, where is possible to see the fragmentation of glycosides by loss of the mass corresponding to rhamnose (≈146 Da).

The peak numbers 4–6 were not identified by the LC-MS and MALDI/MS/MS techniques, but taking into account the UV spectrum of peak 6 (maximum absorbance in the 251–255 nm range) and MW data (250 MW), we hypothesized that this peak could correspond to a low molecular weight substance, such as a polyalcohol or a phenolic acid. Besides, according to Rijke et al. [37], flavonoids display a typical UV spectrum with a first absorbance maximum in the 240–285 nm range and a second one in the 300–550 nm range, as exhibited by quercitrin and myricitrin.

In previous studies, the compounds quercitrin and afzelin were identified in the ethanolic leaf extract from a *Nectandra grandiflora* specimen collected in São Paulo, Brazil [24,36]. Moreover, Ribeiro et al. [24] found protocatechuic acid, a naturally occurring phenolic acid, as constituent of *Nectandra grandiflora* leaves. Other phenolic compounds such as neolignan licarin B [38] and burchellin [24] were isolated from *Nectandra grandiflora* leaves and fruits, respectively.

### 2.4. TG/DTG Profiles

**Figure 3:**
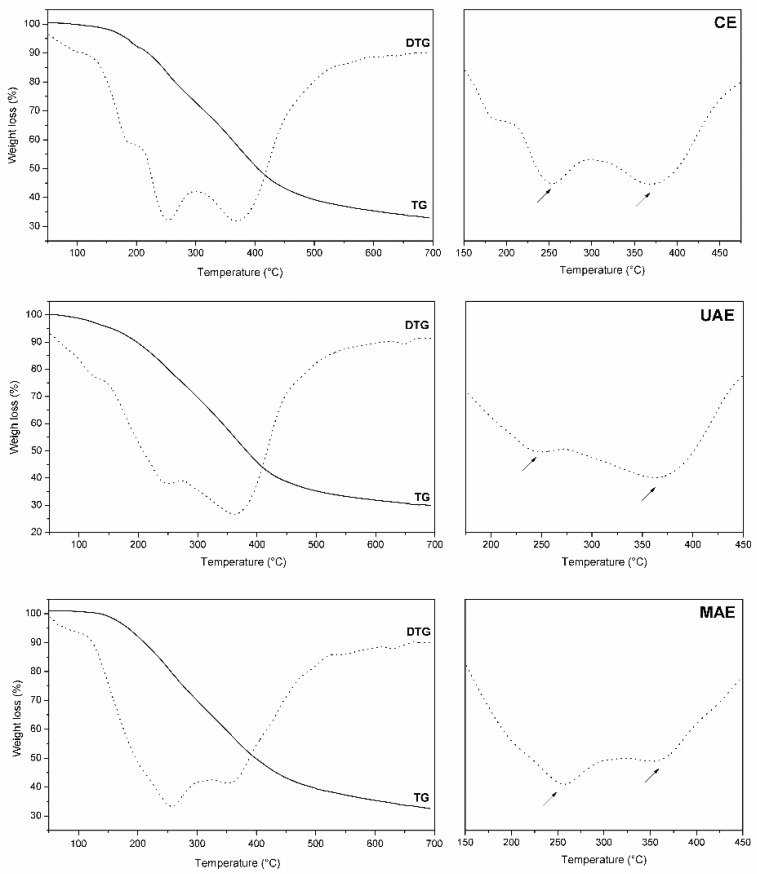
TG/DTG profiles of *Nectandra grandiflora* extracts obtained by different techniques. CE: Conventional Soxhlet extraction; UAE: Ultrasound-assisted extraction; MAE: Microwave-assisted extraction. The arrows indicate the temperatures where the greatest mass loss occurred.

Thermal analysis was carried out as a first step to characterize the decomposition stages and thermal stability of *Nectandra grandiflora* extracts in the absence of parallel reactions. The TG/DTG curves of all extracts exhibited similar decomposition patterns, as displayed in Figure 3. 

During the extraction process, several bioactive substances from different class can be recovered from the plant raw material. Besides phenolic compounds, polar solvents (such as ethanol) can also extract lipids, fats, terpenoids, sugars and chlorophylls [36].

DTG curves from all extracts showed a slight mass loss within the temperature range 50–180 °C, which was mainly caused by water desorption [39] and decomposition of terpenoid derivatives (volatile compounds) [40]. All thermograms showed two main degradation peaks indicating the main organic matter losses [41]. The first one was between 240 and 270 °C (peak 1), and the second between 340 and 380 °C (peak 2). Peak 1 may be attributed to thermal breakdown of aliphatic structures and glycosylated aromatic compounds (such as quercetin-3-*O*-rhamnoside detected by the LC-MS technique), and peak 2 was associated with the degradation of more stable compounds [39,40,41,42,43,44].

At the end of the TG process (about 700 °C), the solid residues for CE, UAE and MAE were 32.76, 29.85 and 31.88%, respectively. The percentage of residual mass in the CE sample can be attributed to the higher presence of phenolic substances (78.66% estimated by LC-MS), which tend to become fixed carbon during the pyrolysis process [45].

### 2.5. Solubility Results

The solubility of the obtained extracts was evaluated using an organic solvent and then analysing the optical images formed by the solutions (Figure S1). We detected similar physical characteristics (solubility) of the obtained extracts. The solution prepared with CE sample presented an average concentration of 3.98 × 106 particles/µL, while UAE sample presented 3.49 × 106 particles/µL and MAE, 3.31 × 106 particles/µL. No pronounced variation in the average diameter of the undissolved particles was observed among the extract solutions, only a slight variation between MAE and CE samples was detected (4.9 µm and 5.1 µm, respectively). The applied method is a novel fast assessment that provides useful information such as particle size and organic solubilization, which are important characteristics in the natural materials subject and regarding environmental issues [46].

### 2.6. Antioxidant Activity

**Figure 4:**
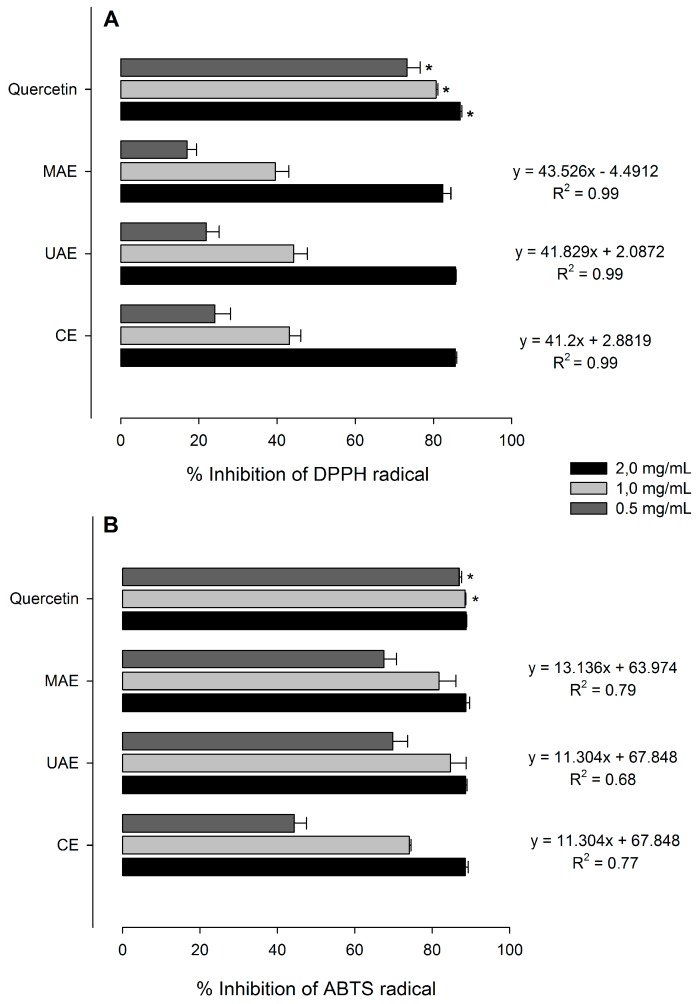
Antioxidant capacities on DPPH (**A**) and ABTS (**B**) free radicals of leaf extracts obtained from *Nectandra grandiflora*. * Indicate significant differences among the extraction methods and quercetin for the same concentration by Tukey test (*p* < 0.05). CE: Conventional Soxhlet extraction; UAE: Ultrasound-assisted extraction; MAE: Microwave-assisted extraction; Quercetin: Positive control.

Regarding the antioxidant activities, *Nectandra grandiflora* leaf extracts were able to inhibit both DPPH and ABTS free radicals, in comparison with quercetin, used as positive control. The antioxidant effect in a concentration-response relationship was verified in all samples and the corresponding equations are displayed in Figure 4.

All ethanolic extracts showed good scavenging activities to reduce the stable radical DPPH to yellow-colored 2,2-diphenyl-1-picrylhydrazine. Besides, the results indicate that there are no significant differences between the *Nectandra grandiflora* extract samples at the same tested concentration. At the highest concentration (2 mg/mL), the DPPH radical inhibition reached 85.59% with CE and UAE, and 82.39% with MAE. Quercetin reached 86.85% of inhibition at 2 mg/mL, significantly different in all *Nectandra grandiflora* samples. In the ABTS radical cation decoloration assay, the leaf extracts have a similar inhibition rate to that found against DPPH radical. No statistical differences among any of the samples were detected at 2 mg/mL (Figure 4B). At the lowest concentration, UAE presented better values than the CE and MAE procedures; however, these values were lower than those achieved by the positive control was. Considering the *R*^2^ values among the tests, the observed differences can be due to the reaction environment (alcoholic or hydro-alcoholic), the solubility of the main compounds in each reaction medium as well as the variable activity of antioxidants in reducing the pre-formed radical cation radical (ABTS^•+^) to ABTS [47].

Our findings illustrate the antioxidant capacity of *Nectandra grandiflora* leaves was weakly affected by the extraction protocol [17]. The positive results detected can be assigned to the phytochemicals present in the leaf extracts. Probably the most active natural phytochemicals in the *Nectandra grandiflora* extracts are from the flavonoid chemical class, since they present specific structural characteristics that promote antioxidant activity. The *o*-catechol group on the B-ring as occurs in quercetin derivatives, the major phytochemicals detected in this study, is the most important of them [48]. Moreover, the same partial structure appears in protocatechuic acid [49], already described in *Nectandra grandiflora* [24]. Other flavonoids with chemical characteristics can contribute the antioxidant properties, such as the three hydroxyl groups on the B-ring (present in myricetin derivatives) and the α,β-unsaturated carbonyl system on the C-ring [45]. These characteristics confer great stability to the phenolic radical as soon it is formed after one H radical donation to DPPH [24,50,51].

### 2.7. Antifungal Activity

**Figure 5:**
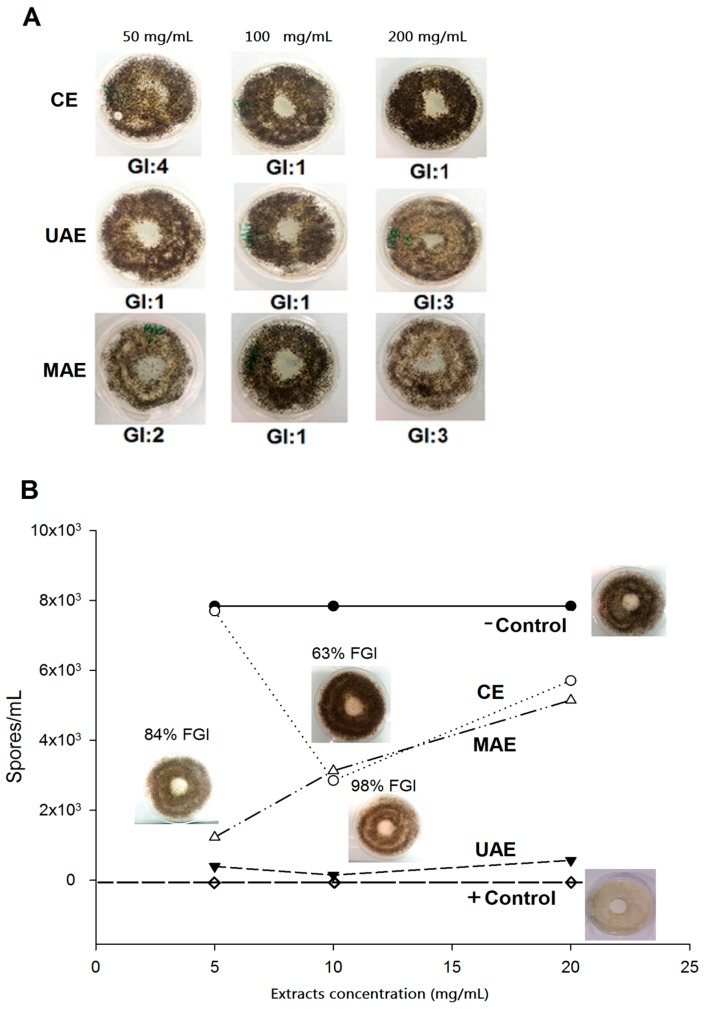
Antifungal activity of *Nectandra grandiflora* extracts against *Aspergillus niger* by the potato dextrose agar method (**A**) and cellulose pellets method (**B**). GI: Growth intensity; FGI: Fungal growth inhibition; CE: Conventional Soxhlet extraction; UAE: Ultrasound-assisted extraction; MAE: Microwave-assisted extraction.

The methods used to measure the antifungal effect of the extracts were designed to determine both the efficacy of compounds to prevent fungal growth and as a method to assess the susceptibility of the growth of molds to impregnated materials. Results from exposure of *Aspergillus. niger* to various concentrations of *Nectandra grandiflora* extracts are displayed in Figure 5, which in turn were contrasted with a positive control (amphotericin B) and with a negative control (without product).

As indicated in Figure 5, the leaf extracts did not inhibit fungal growth dose-dependently according to the evaluated methods. In Figure 5A, all extracts tested at 100 mg/mL of concentration were effective in controlling the fungal growth (growth intensity = 1), by visual assessment (without contact with the center of the dish).

At 200 mg/mL, the samples of CE were more efficient than those samples from UAE and MAE (growth intensity = 3). A similar trend of fungal growth inhibition was observed with the second method (cellulose pellets; Figure 5B). The CE sample at 100 mg/mL inhibited 63% of fungal growth and UAE extract reached to a maximum of 98% at the final concentration of 100 mg/mL, compared to non-treated control. Regarding the MAE sample, higher fungal inhibition (84%) was achieved at a concentration of 50 mg/mL.

To the best of our knowledge, there are no reports regarding the inhibitory potential of *Nectandra grandiflora* against *Aspergillus niger*. Previously, the antifungal activity of the *Nectandra grandiflora* leaf essential oil against wood-rot fungi was reported [40], where it was able to inhibit *Pycnoporus sanguineus* and *Gloeophyllum trabeum* growth at a concentration of 5 µl/mL. From these findings and from Magro et al.’s study [52], which examined the *Anthemis nobilis* leaf extract and found an inhibitory effect at 920 mg/mL against *Aspergillus niger*, we can affirm that the *Nectandra grandiflora* leaves possess potential antifungal constituents.

## 3. Materials and Methods

### 3.1. Plant Material

Aerial parts of *Nectandra grandiflora* were collected from a natural habitat in Jaguari County, in the South of Brazil (29°26′ S and 54°40′ W), in December 2013. Leaves were separated from the branches and fractionated in order to achieve the ethanolic extracts. A voucher specimen, identified by Solon Jonas Longhi, is archived under number 13162 at the Herbarium of Biology Department (SMDB, Federal University of Santa Maria, Brazil).

### 3.2. Preparation of Ethanolic Extracts

Leaves were air-dried at room temperature (25 °C), milled (Willey mill, Swedesboro, NJ, USA) and then extracted with ethanol 96%, which is regarded as a generally recognized as safe (GRAS) solvent, at a raw material: solvent ratio of 1:20, by means of ultrasound-assisted extraction (UAE), microwave-assisted extraction (MAE) or conventional solvent extraction (CE) [17]. The extraction procedures were as follows: the UAE was carried out using an ultrasonic cleaner (Elmasonic S 70H, Elma Schmidbauer GmbH, Singen, Germany) at a power of 750 W and 50 °C and MAE was done with a CEM Discover microwave (CEM Corporation, Matthews, NC, USA) at 50 °C and power controlled by the equipment. Both UAE and MAE were performed for 30 min using ca. 5 g per replicate (*n* = 3). The CE was performed using a Soxhlet apparatus (Hermanos Álamo, Madrid, Spain) (ca. 15 g per replicate; *n* = 3) until the total exhaustion of the plant material (24 h).After the extraction period, the ethanolic extracts were cooled to room temperature and filtered. The solvent was removed at 50 °C under reduced pressure on a rotary evaporator and then the extraction yields were calculated by weighing the extracts obtained per each 100 grams of dried plant based on dried weight (DW).

### 3.3. Total Phenolic Content

The total phenolic content of *Nectandra grandiflora* leaf extracts was measured spectrophotometrically (Jasco V-630 spectrophotometer, Jasco Deutschland GmbH, Hamburg, Germany) by the Folin-Ciocalteu’s method, as described by Cândido et al. [53], with some modifications. Dried extracts were solubilized in methanol (0.5 mg/mL), aliquots of these samples (0.25 mL) were mixed with 2.5 mL of distilled water, and 0.25 mL of the Folin-Ciocalteu reagent (previously diluted 1:10 with distilled water). After 5 min, 0.25 mL of sodium carbonate (75 mg/mL in aqueous solution) was added and adjusted to 10 mL with distilled water. The mixtures were kept at room temperature for 60 min and the absorbance was measured at 725 nm. Gallic acid (0–0.2 mg/mL) was used for calibration of a standard curve. The calibration curve was linear at *R*^2^ = 0.99, and the results were expressed as mg of gallic acid equivalents per gram of dried weight (mg GaE/g DW). Triplicate measurements were taken and data were presented as mean ± standard deviation.

### 3.4. Flavonoid Content

The flavonoid content of the extracts was determined by the AlCl_3_ technique [54] using a spectrophotometer (Jasco V-630). The results were expressed as mg of quercetin equivalents (QE) per g DW from a standard calibration curve (0–0.1 mg/mL; *R*^2^ = 0.99).

### 3.5. Infrared Analysis

In order to determine the functional groups presents in CE, UAE and MAE samples, Fourier Transform Infrared (FTIR) analysis was applied. Infrared spectra were recorded in a Perkin Elmer spectrophotometer (Waltham, MA, USA) at a resolution of 4 cm^−1^ over the 700–4000 cm^−1^ range using milled samples [55].

### 3.6. LC-UV/ESI-HR-MS Analysis

LC-UV/ESI-HR-MS analysis was carried out on a UPLC system (Waters ACQUITY UPLC System, Milford, MA, USA) equipped with a UV-Vis photodiode array detector and coupled to a mass spectrometer. UV spectra were recorded between 200 and 500 nm and the UV detection was measured at 280 nm (100% correspond to sum of area of six detected peaks in each sample). An Acquity C_18_ column (100 × 2.1 mm i.d., 1.7 µm) at 40 °C was used to chromatography separation. The mobile phase was constituted by two solvents: water-formic acid (0.1%, A) and methanol (B), and the gradient elution had the following profile: 0–25 min 95% A, 25–27.7 min 1% A and 27.7–30 min 5% A at a flow rate of 300 µL/min. Extract samples were prepared at 200 µg/mL in methanol:water (1:1) and 10 µL aliquots were injected for analysis.

Mass spectra were acquired using a LCT Premier XE (Waters) equipped with an electrospray ionization (ESI) source operated in the positive W mode. The experimental parameters were set as follows: the capillary voltage was 750 V; cone voltage was 50 V; and ions were recorded in the range of *m*/*z* 100–1000. In order to obtain exact mass measurements, leukine-enkephalin was used as lockmass reference compound (*m*/*z* 556.2771). Data acquisition and analysis were performed using Waters MassLynx 4.1 software (Waters Corporation, Milford, MA, USA). 

### 3.7. MALDI-TOF/TOF MS Analysis

MALDI-TOF/TOF mass analysis were performed on an Ultraflextreme III time-of-flight mass spectrometer equipped with a pulsed Nd:YAG laser (355 nm) and controlled by FlexControl 3.3 software (Bruker Daltonics, Bremen, Germany). The acquisitions (total of 4000–5000) were carried out in positive reflector ion mode with pulse duration of 70 ns, laser fluence of 35% and laser frequency of 1 kHz. Laser intensity was set marginally above the threshold of ionization to avoid fragmentation (less than 10% for all the cases). Fragmentation of the molecules were performed with a LIFT cell voltage of 19 kV and a final acceleration voltage set at 29.3 kV and the parent mass ions were assigned manually (monoisotopic peak M + Na). Ion source 1, 2 and lens voltages were set at 7.56, 6.86 and 3.52. 5 μL of sample (extract at 200 µg/mL in Water/MeOH) was mixed with 10 μL of α-Cyano-4-hydroxycinnamic acid matrix solution (10 mg/mL in Methanol Water 1/1). 1 µL of the analyte/matrix mixture was deposited onto the polished stainless-steel MALDI target plate and was allowed to dry. 

All the peaks were detected as sodium/potassium adducts. The acquired data was processed (baseline substraction and normalized) using the Bruker FlexAnalysis 3.3 software (Bruker Daltonics, Bremen, Germany).

### 3.8. Thermogravimetric Analysis

Thermal behavior of *Nectandra grandiflora* extracts (CE, UAE and MAE) was measured in a nitrogen atmosphere using a TGA/SDTA RSI analyser (Mettler Toledo, L’Hospitalet de Llobregat, Barcelona, Spain) according to Herrera et al. [55]. For the quantitative calculations, the response factors between the weight gain (TG) and the mass loss rate (DTG) were determined.

### 3.9. Solubility Measurement of Extracts

To investigate the extracts solubility in organic solvents, CE, UAE and MAE samples were diluted in dimethyl sulfoxide (DMSO) at a concentration of 10 mg/mL and the solubility was analyzed by a Cellometer^®^ Mini Vision equipment (Nexcelom Bioscience LLC, Lawrence, MA, USA). Each extract solution was precisely pipetted (0.02 mL) into a Nexcelom disposable counting chamber to determinate the concentration of undissolved extracts (particles/µL) and the particles sizes (average size from 1 to 35 µm) by the Cellometer^®^ Mini Counter Software (Nexcelom Bioscience LLC, Lawrence, MA, USA, Software version 1.2.3.3).

### 3.10. Antioxidant Activities

#### 3.10.1. DPPH Assay

The DPPH (2,2-diphenyl-1-picrylhydrazyl) radical scavenging activity of the extracts was carried out according to Dudonné et al. [56] on a Jasco V-630 Spectrophotometer. An aliquot (0.02 mL) of leaf extracts at different concentrations was added to 2 mL of DPPH methanolic solution (0.06 mM) and kept at room temperature for 30 min. The absorbance was measured at 517 nm and quercetin was utilized as positive control.

#### 3.10.2. ABTS Assay

The antioxidant capacity was also evaluated by ABTS spectrophotometric assay [47]. Extract samples were diluted in methanol at different concentrations and an aliquot (0.04 mL) was added to 2 mL of ABTS radical solution. This solution was prepared by mixing ABTS (7 mM) and potassium persulfate (2.45 mM) in water for 12–16 h at room temperature in a light-free environment. Then, the absorbance of radical solution was adjusted to 0.60 ± 0.02 at 734 nm in ethanol: water (1:1). Each extract sample was measured between 1 and 6 min.

### 3.11. Determination of Antifungal Activity against Aspergillus niger

The fungus *Aspergillus niger* (Tiegh MB284309 CBS-KNAW, Utrecht, The Netherlands) was cultured on potato dextrose agar (PDA) for 7 days at 27 ± 1.5 °C and used in this assay. Extracts samples were diluted in DMSO at final concentrations of 50, 100 and 200 mg/mL, and then evaluated by two methods: (1) extracts pipetted directly in PDA medium and inoculated with fungal strain; and (2) extracts impregnated in cellulose pellets and exposed to fungal strain in PDA.

In the first method, an aliquot (40 μL) of each extract was pipetted to the center of a Petri dish filled with PDA, and around that was inoculated a fungal strain. The Petri dishes were sealed and incubated at 27 ± 1.5 °C (Selecta Medilow climatic chamber, JP Selecta S.A., Barcelona, Spain) for 7 days. After incubation time, we determined the growth intensity (GI) by visual assessment using a numerical scale according to ISO 846, as displayed in Table 3. Three repetitions of each extracts and control (without extracts) were prepared.

In parallel, cellulose pellets (ø = 10 mm) were soaked with 5 µL of each extract set and placed on Petri dishes with PDA (10 mL) and 0.4% streptomycin. Each PDA dish was inoculated with a spore suspension (1 × 10^6^ spores/mL) and incubated at 27 ± 1.5 °C for 7 days. Subsequently, pellets were removed from Petri dishes and washed with sterile Ringer’s solution (Sigma-Aldrich-96724, St. Louis, MI, USA). The solution was stained (Lactophenol blue) and homogenized to count the spores concentration on the pellets with a Cellometer^®^ Mini automated cell counter by placing 20 µL of each spore solution inside counting chambers and using Cellometer^®^ Mini software for the analysis. The fungal growth inhibition (FGI %) was calculated as concentration of spores (conidia) per mL, according to the following Equation (1):

(1) $$\mathrm{FGI}\;(\%)=\frac{Cg-Tg}{Cg}\times 100$$

where, *Cg* is the average spores concentration in the control sample and *Tg* is the average concentration in the treated one [57].

### 3.12. Statistical Procedure

The results are expressed as the mean of three measurements ± standard deviation. Normality (Shapiro-Wilk) and Equal Variance (Levene) tests were performed before the statistical approach. Analysis of variance (ANOVA) was conducted for the values of total phenolic, flavonoid and tannin contents, as well as for antioxidants data, followed by Tukey test. The differences with *p* < 0.05 were considered significant.

## 4. Conclusions

The experimental results indicated that ultrasound- and microwave-assisted extraction techniques were effective to recover bioactive compounds from *Nectrandra grandiflora* leaves. Despite the fact that higher phytochemical contents were achieved by conventional extraction, the chemical composition, thermal stability and antioxidant activity did not present great differences to that found with the alternative green techniques. Besides, microwave- and ultrasound-assisted are timesaving extraction processes with lower energy consumption comparing to the Soxhlet method; however, it is necessary to optimize the ultrasound and microwave process conditions to increase the phytochemical yields. Furthermore, *Nectandra grandiflora* by-products could be an interesting source of active compounds for the natural antioxidants and antifungal market.

## Figures and Tables

**Table 1:** Effect of extraction method on the phytochemical yields, total phenolic (TPC) and flavonoid (FLC) contents of *Nectandra grandiflora* Nees leaf extracts.

Extraction		TPC (mg GaE/g DW)	FLC (mg QE/g DW)
Method	Yield (g DW/100g Dried Plant)		
CE	22.16 ± 1.18 ^a^	279.00 ± 7.32 ^a^	150.85 ± 0.71 ^a^
UAE	13.99 ± 2.58 ^b^	254.94 ± 7.58 ^b^	114.50 ± 0.71 ^b^
MAE	8.21 ± 2.74 ^c^	229.62 ± 1.85 ^c^	123.83 ± 3.60 ^b^
F	28.32	62.55	22.40
*p*	<0.001	<0.001	0.002
MSD	2.28	10.18	5.47

Lower case letters indicate significant differences among the extraction methods for the same column by Tukey test (*p* < 0.05). CE: Conventional Soxhlet extraction; UAE: Ultrasound-assisted extraction; MAE: Microwave-assisted extraction; DW: Extract based on dried weight; GaE: Equivalent gallic acid; QE: Equivalent quercetin; MSD: Minimum Significant Difference.

**Table 2:** Phenolic compounds detected in the leaf extracts obtained from *Nectandra grandiflora* Nees by LC-UV/ESI-HR-MS in the positive mode.

**CE**							
**Proposed Compound**	**Peak**	**t_R_ (min)**	**λ_max_ (nm)**	**MW**	**[M + Na]^+^ (*m*/*z*)**	**Fragment Ions (*m*/*z*)**	**Peak Area (%)**
Myricetin-rhamnoside	**1**	10.17	256.93; 351.93	464	487.1861	**319.1163**; 273.2263	11.26
Quercetin-rhamnoside	**2**	11.72	255.93; 349.93	448	471.1797	**303.1064**; 325.1030	65.32
Kaempferol-rhamnoside	**3**	12.96	263.93	432	455.1926	**218.2257**; 287.1182; 304.2944	5.13
Unidentified	**6**	19.98	253.93	250	273.2414	**219.3682**; 149.1013; 137.0943	15.57
Total identified							*81.71*
**MAE**							
**Proposed Compound**	**Peak**	**t_R_ (min)**	**λ_max_ (nm)**	**MW**	**[M + Na]^+^ (*m*/*z*)**	**Fragment Ions (*m*/*z*)**	**Peak Area (%)**
Myricetin-rhamnoside	**1**	10.14	258.93; 352.93	464	487.1981	**319.1164**; 273.2266; 341.1031	9.95
Quercetin-rhamnoside	**2**	11.69	255.93; 340.93	448	471.1819	**303.1085**; 325.1035	62.54
Kaempferol-rhamnoside	**3**	12.93	263.93	432	455.1905	**287.1170**; 218.2304; 309.1042	5.50
**Unidentified**	**6**	**19.98**	**254.93**	**250**	**273.2419**	**219.3695; 149.1015**	**19.58**
Total identified							*77.99*
**UAE**							
**Proposed Compound**	**Peak**	**t_R_ (min)**	**λ_max_ (nm)**	**MW**	**[M + Na]^+^ (*m*/*z*)**	**Fragment Ions (*m*/*z*)**	**Peak Area (%)**
Myricetin-rhamnoside	**1**	10.14	256.93; 348.93	464	487.1881	**319.1158**; 273.2258; 341.1027	9.77
Quercetin-rhamnoside	**2**	11.69	255.93; 349.93	448	471.1833	**303.1102**; 325.1038	61.18
Kaempferol-rhamnoside	**3**	12.93	263.93	432	455.1806	**287.1170**; 218.2305; 304.2939	5.11
Unidentified	**6**	19.98	255.00	250	273.2429	**220.2213**; 149.1024; 137.0951	20.83
*Total identified*							*76.06*

The base peaks are in bold; CE: Conventional Soxhlet extraction; UAE: Ultrasound-assisted extraction; MAE: Microwave-assisted extraction.

**Table 3:** Visual assessment of growth intensity according to ISO 846.

Growth Intensity (GI)	Evaluation
0	No growth apparent under magnification
1	No visible growth but visible under magnification
2	Visible growth up to 25% coverage
3	Visible growth up to 50% coverage
4	Visible growth up to 75% coverage
5	Heavy growth covering more than 75% of the studied area

## Supplementary Materials

The Supplementary Materials are available online.
