# Supplementary material for: Nectandra grandiflora By-Products Obtained by Alternative Extraction Methods as a Source of Phytochemicals with Antioxidant and Antifungal Properties
Source: Molecules. 2018 Feb 9;23(2):372. doi: 10.3390/molecules23020372 (PMC6017794; doi:10.3390/molecules23020372)
Supplement: Supplementary file 1 [file molecules-23-00372-s001.docx]

**Supplementary Materials**

*Nectandra grandiflora* By-Products Obtained by Alternative Extraction Methods as a Source of Phytochemicals with Antioxidant and Antifungal Properties

Daniela Thomas da Silva ^1^, Rene Herrera ^2^, Berta Maria Heinzmann ^3^, Javier Calvo ^4^ and
Jalel Labidi ^2,^*

^1^ Center of Rural Sciences, Federal University of Santa Maria, Ave. Roraima 1000, Santa Maria 97105-900, Brazil; dthomasdasilva@gmail.com

^2^ Biorefinery Processes Research Group, Chemical and Environmental Engineering Department, University of the Basque Country (UPV/EHU), Plaza Europa 1, 20018 Donostia, Spain;
reneherdiaz@hotmail.es or renealexander.herrera@ehu.eus

^3^ Department of Industrial Pharmacy, Federal University of Santa Maria, Ave. Roraima 1000, Santa Maria 97105-900, Brazil; berta.heinzmann@gmail.com

^4^ Chromatography and Mass Spectrinetry Platform, CIC BiomaGUNE, Paseo Miramon 182,
200009 San Sebastian, Spain; jcalvo@cicbiomagune.es

***** Correspondence: jalel.labidi@ehu.es; Tel.: +34-94301-7178

**Table S1:** Peaks and assignments of FTIR spectra of ethanolic extracts from *Nectandra grandiflora* Nees leaves.

| **Main vibrational bands of extracts (wavenumbers, cm^-1^)** | | | **Assignment** | **Peak**  **number^a^** |
| --- | --- | --- | --- | --- |
| **CE** | **UAE** | **MAE** |  |  |
| 3314 | 3321 | 3322 | –OH stretching vibration | 1 |
| - | 2945 | 2946 | Methylene asymmetrical stretching vibration | 2 |
| 2917 | 2917 | 2918 | Methylene asymmetrical stretching vibration | 3 |
| 2849 | 2849 | 2850 | Methylene/methyl symmetrical stretching vibration | 4 |
| 1709 | 1710 | 1709 | C=O stretching vibration in acids | 5 |
| 1605 | 1606 | 1606 | Within-ring skeletal bands | 6 |
| 1515 | 1515 | 1515 | Aromatic C=C–C stretching vibration | 7 |
| 1443 | 1444 | 1444 | Aromatic ring vibration | 8 |
| 1361 | 1374 | 1375 | OH in-plane bending in primary and secondary alcohols | 9 |
| 1275 | 1277 | 1271 | C–O stretch of pyran ring | 10 |
| 1199 | 1199 | 1199 | C-OH deformation in phenols | 11 |
| 1164 | 1164 | 1165 | Aromatic CH in-plane bending vibration | 12 |
| 1059 | 1043 | 1046 | C–O stretching vibration | 13 |
| - | 878 | 879 | Aromatic CH out-of-plane bending vibration | 14 |
| 815 | 816 | 816 | Aromatic CH out-of-plane bending vibration | 15 |
| ^a^ Peak numbers refer to the assignments in Figure 1. | | | | |

*
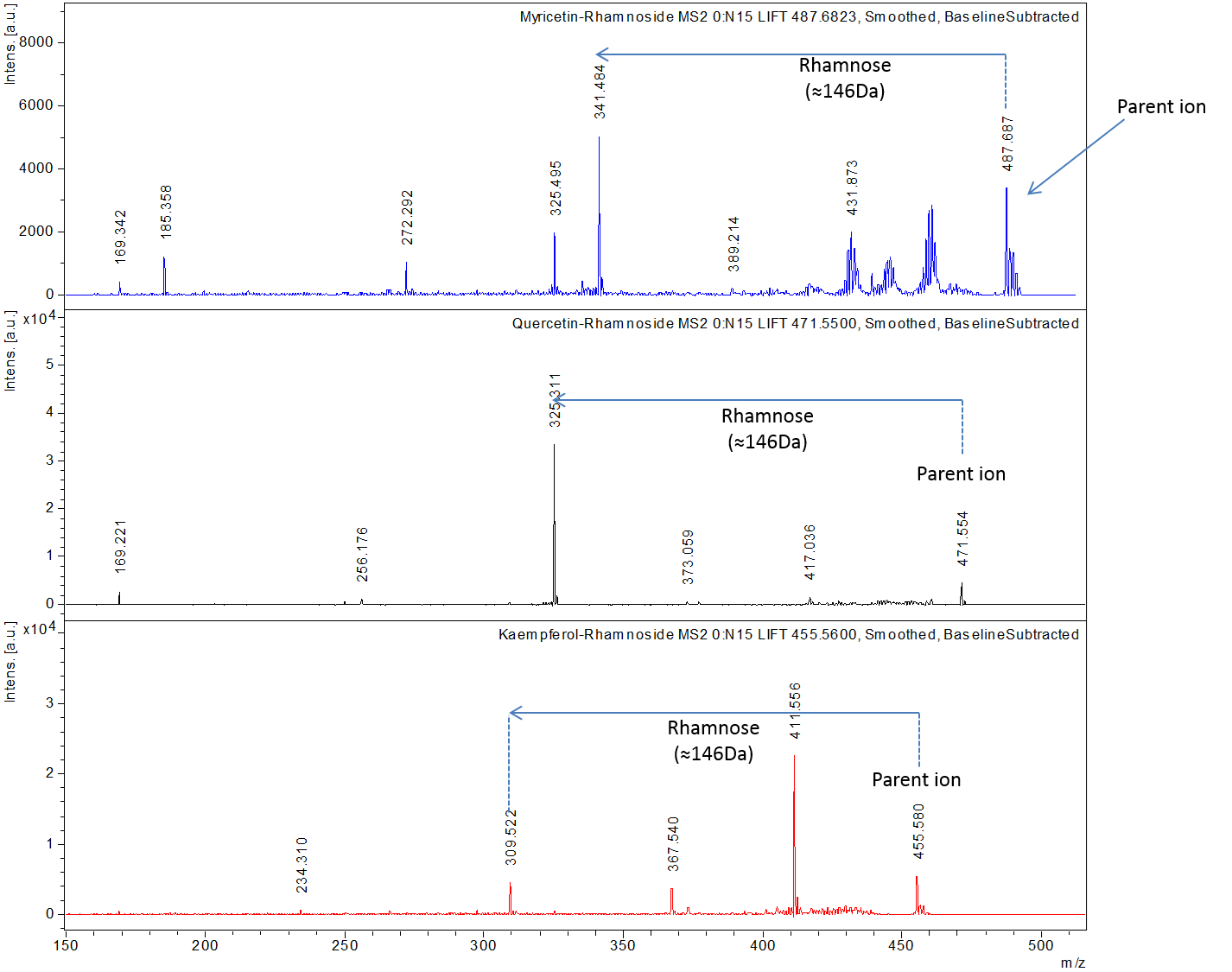
*

**Figure S1:** MS fragmentation of the peaks 1, 2 and 3 by MALDI/MS/MS analysis.


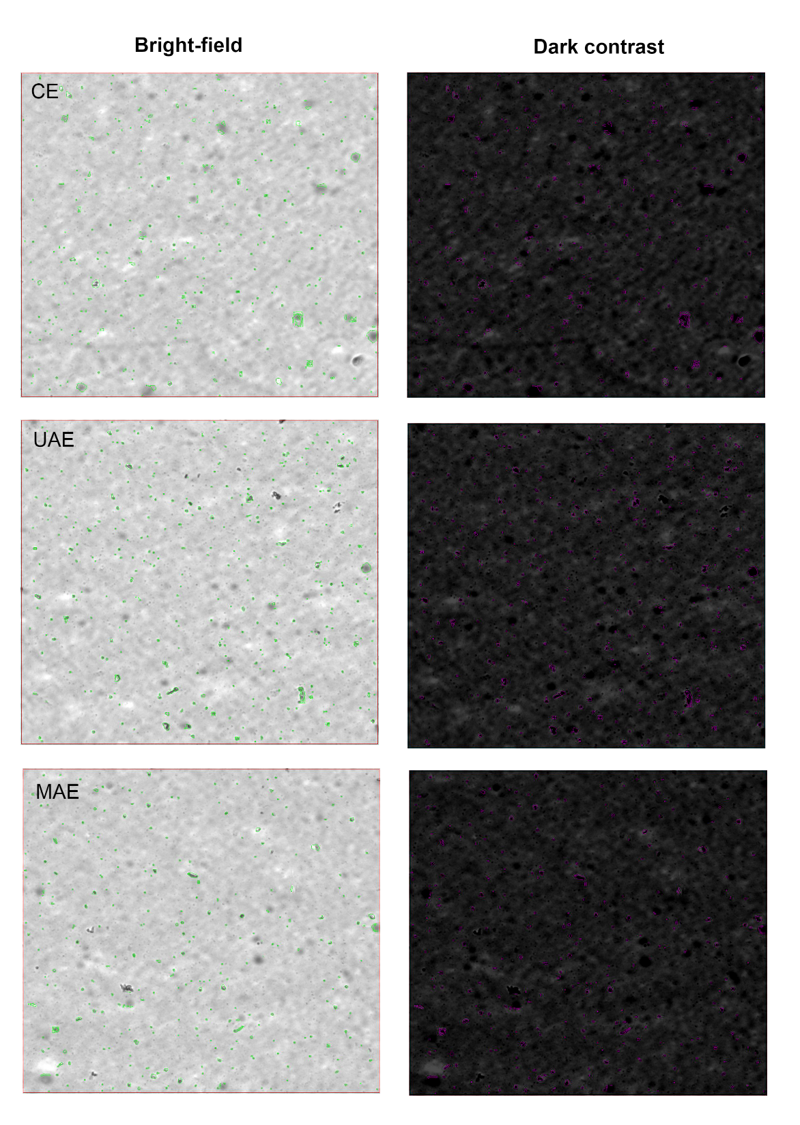


**Figure S2:** Solubility measurement of *Nectandra grandiflora* extracts using DMSO as solvent.
